# L-kynurenine or nicotinamide supplementations mitigate uterine decidualization impairments during early pregnancy of uninephrectomized mice

**DOI:** 10.3389/fphys.2026.1774244

**Published:** 2026-03-10

**Authors:** Yuye Wang, Qing Ma, Meitong Chen, Yukako Kayashima, Nobuyo Maeda-Smithies, Feng Li

**Affiliations:** Department of Pathology and Laboratory Medicine, The University of North Carolina, Chapel Hill, NC, United States

**Keywords:** endothelin-1, L-kynurenine, mice, nicotinamide, unilateral nephrectomy

## Abstract

Impaired renal reserve induced by unilateral nephrectomy (UNx) leads to the full-spectrum of phenotypes of preeclampsia (PE) in mice. L-kynurenine (a product of L-tryptophan metabolism) supplementation throughout the entire pregnancy rescues the PE-like phenotype in mice. However, whether UNx dams have endometrial decidualization deficiency during early pregnancy and whether L-kynurenine supplementation improve decidualization in UNx dams are not clear. While our prior study showed that nicotinamide (NAM) supplementation has beneficial effects on decidualization in mice with excess endothelin 1 (ET-1), whether NAM supplementation has a beneficial role in UNx dams is unknown. Here, we tested our hypothesis that UNx impairs uterine decidualization and L-kynurenine/MAM treatment improves decidualization. We compared implantation sites between sham dams, UNx dams, and UNx dams treated with L-kynurenine or NAM. Implantation sites of UNx dams had lagged embryos with distorted ectoplacental cone and reduced vascular density in mesometrial regions of deciduae. The decidual expression of VEGF-A and markers of decidualization (e.g., BMP2, prolactin) was decreased in UNx dams. Both L-kynurenine and NAM corrected the abnormality present in maternal-embryo interface of UNx dams. In addition, the decidual expression of ET-1 and its type B receptor decreased in UNx dams, and L-kynurenine/NAM increased their expression in the deciduae. Our results show that suppressed decidual ET-1/EDNRB signaling could play a role in the poor decidualization (decreased BMP2/prolactin) and angiogenesis (decreased VEGF) in UNx dams and both L-kynurenine and NAM have potential to improve embryo implantation and subsequent pregnancy outcomes.

## Introduction

Preeclampsia (PE) is a pregnancy associated hypertensive disorder affecting 5%–8% of all pregnancies. The incidence of PE has been increasing in the past three decades. The etiology of PE is largely unknown and there is no real treatment for this human disease: the ultimate treatment is the delivery of placenta and infant (Turbeville and Sasser, 2020; Rana et al., 2019).

Although the etiology of PE is largely unknown, the maternal pre-existing conditions including obesity, diabetes mellitus and kidney problems play an important role in the pathogenesis of PE (Roberts et al., 2011). The human studies have reported that live kidney donors have increased risk for PE (Garg et al., 2015; Ibrahim et al., 2009; Reisaeter et al., 2009). Recently, Dupont et al. reported that wild type (WT) mice with impaired renal reserve induced by unilateral nephrectomy (WT-UNx) develop the full-spectrum of the PE-like phenotype including elevated blood pressure (BP) and urinary albumin excretion and increased fetal resorption accompanied with placental pathologies after 2 weeks of gestation (Dupont et al., 2022). The levels of progesterone and estradiol are reduced in preeclamptic women (Wan et al., 2018). These two hormones are altered in kidney transplant recipients (Kim et al., 2012), while the report of their levels in pregnant women with one kidney is scarce and it is not clear the levels of the hormones in UNx pregnant mice.

Appropriate establishment of maternal uterine decidua, decidual angiogenesis and vascular remodeling is critical for placental development, thus, healthy pregnancy. If the decidual process is disturbed, the pathologies of pregnancy happen, including PE. For example, we have demonstrated that female mice having 2-3x circulating endothelin-1 (ET-1) levels than WT females have insufficient endometrial decidualization and angiogenesis leading to impaired trophoblast cell differentiation and invasion in early pregnancy and subsequent the manifestation of the PE-like phenotypes in late pregnancy (Wang et al., 2025; Li et al., 2018). However, whether WT-UNx dams have impaired decidualization resulting in problematic maternal-embryo interaction in early pregnancy which leads to a PE-condition later observed in these mice is unknown.

L-kynurenine is a naturally occurring metabolite of the amino acid L-tryptophan. Dupont et al. have also demonstrated L-kynurenine supplementation throughout the entire pregnancy rescued the symptoms of PE in WT-UNx mice (Dupont et al., 2022). L-kynurenine improved placental perfusion and spiral artery remodeling in association with decreased placental *sFlt1* mRNA levels and improved labyrinthine vasculature (Dupont et al., 2022). Prior studies show that L-tryptophan metabolites are important for early placentation and vascularization. (Munn et al., 1998; Broekhuizen et al., 2021). L-tryptophan serves as a precursor for several important metabolites, including nicotinamide and kynurenine pathway is crucial for the body’s ability to synthesize nicotinamide (Fukuwatari and Shibata, 2013).

Nicotinamide (amide form of vitamin B3, NAM) is a potent inhibitor of ET-1 downstream of ADP ribosylcyclase (Thai and Arendshorst, 2008; Thai et al., 2007). We have tested this agent in two different mouse models of PE [one with experimentally induced sFLT1 overexpression and another with genetically lacking ASB4 (Ankiryn-repeat-and-SOCS-box-containing-protein (Townley-Tilson et al., 2014)], and reported that NAM treatment throughout the entire pregnancy decreases blood pressure (BP), renal injury and prolongs pregnancy. NAM ameliorates a PE-like condition in mice with reduced uterine perfusion pressure (RUPP) as well (Fushima et al., 2017). Thus, NAM has the potential to reverse serious maternal sequelae of PE to allow prolongation and improve infant outcomes. In addition, our most recent work shows that NAM improves the decidualization and angiogenesis which are impaired in dams with excess ET-1 (Wang et al., 2025). However, whether and how NAM supplementation could prevent the potential problems in the early pregnancy in UNx dams has not been investigated.

Accordingly, in the current study, we investigated 1) the role of impaired renal reserve in endometrial decidualization and angiogenesis in early pregnancy when implantation occurs using WT-UNx mice, 2) whether supplementation of L-kynurenine or NAM exerts any beneficial effects in decidualization and angiogenesis in WT-UNx mice.

## Materials and methods

Mice: Wild type (WT) mice (C57BL/6J) including both sexes were housed in standard cages on a 12 h light/dark cycle and were allowed free access to food and water. All experiments were carried out in accordance with the National Institutes of Health guideline for use and care of experimental animals, as approved by the IACUC of the University of North Carolina at Chapel Hill (protocol #24–181).

Uninephrectomy (UNx): Surgery was performed in 10 -week-old WT female mice. Mice were randomly enrolled into either sham or uninephrectomy surgery. The animals will be anesthetized with 1%–2% of isoflurane (IsoThesia, Henry Schein, Melville, NY). For the surgical procedure, hair was removed by a depilatory cream (NairTM Church & Dwight Co. Trenton, NJ) on small areas of the torso (dorsal side) just above the kidneys. The mouse was then moved to the surgical area, and an appropriate level of anesthesia checked (toe pinch). A surgical drape was used to isolate the surgical site followed by a scrub with betadine, and then 75% ethanol for three times. A 0.5 cm incision was made in the skin over the left kidney. Then 0.1% lidocaine, buprenorphine (0.05 mg/kg) and meloxicam (5 mg/kg) were administered s. c. A small incision was made in the muscle fascia, after which the kidney was externalized. The adrenal gland was dissected away from the anterior pole. The renal pedicle was tied off with a suture followed by removal. The muscle fascia layer was sewn shut with 2 interrupted absorbable sutures. The skin was closed using wound clip. During the surgery procedure the mouse was temperature regulated with a heating lamp to maintain a monitored (rectal thermometer) body temperature range between 37 °C and 38 °C (Wang et al., 2017). For sham mice, all steps were same except without tie the renal pedicel and removal of a kidney.

Matings and treatments: Two weeks after surgery, three female mice and one WT male were housed together for overnight (4:00 p.m. to 7:30 a.m.). Females were checked for vaginal plugs each morning, and the day of plug detection was designated as 0.5 days post coitus (dpc) (Li et al., 2018). L-kynurenine or NMA was administered via drinking water at dose of 25 mg/L (approximately 4.2 mg/kg/day) (L-kynurenine, #50–178-9087, ThermoFisher) or 3 g/L (approximately 500 mg/kg/day) (NAM, #72340-100G, Millipore-Sigma), respectively starting at 0.5 dpc for 7 days (Dupont et al., 2022; Wang et al., 2025).

Ultrasonography (HFU): At 7.5 dpc, dams were anesthetized with 2% isoflurane; abdominal hair was removed with depilatory cream. Implantation site detection and location within each uterine horn were visualized transabdominally using the VisualSonics VevoF2 Imaging System with 570s scan head (FUJIFILM VisualSonics Inc., Toronto, ON) at 55 MHz. The 3D mode was used for advanced data acquisition and analysis, with virtual sections obtained in all directions (x-, y-, z- and other plane variations). Scan distance was set at 10.2 mm, with a step size of 0.152 mm and a total of 133 frames were captured per 3D scan. Each ultrasound was finished within ∼15 min; heart rate and body temperature were monitored as we described previously (Wang et al., 2025).

Morphological examination: The implantation sites of 7.5 dpc were examined visually, and the number of them was counted. Fixed implantation sites tissues (4% paraformaldehyde) were sectioned (5 μm) and stained with hematoxylin and eosin (H&E).

Biochemical analyses: The ELISA kit for measuring ET-1, VEGF, and BMP2 was purchased from R&D Systems, Inc. The ELISA kit for estrogen was from abcam (#ab285291, abcam), and progesterone was from CrystalChem (#80559).

Decidua preparation: One implantation site (7.5 dpc) from each dam of four groups was randomly collected. The embryo and ectoplacental cone were carefully removed and the remaining maternal decidua (illustrated in Supplementary Figure S1) was subjected to either RAN isolation as described below (Quantitative RT-PCR section) or homogenization in 0.5 mL buffer (0.1% Triton in PBS) for further ELISA assay (Wang et al., 2025).

Immunohistochemistry (IHC): Application Solutions Kit (#13079, Cell Signaling) was used following the company instructions. In brief, tissue containing paraffin blocks were cut into 5 μm thick. After deparaffinized and hydrated, antigen retrieval was performed in 10 mM sodium citrate buffer (pH = 6.0) and maintain at a sub-boiling temperature for 10 min, followed by cooling on bench top for 30 min. The sections were subsequently incubated with 3% hydrogen peroxide to block endogenous peroxidase and with animal-free blocking buffer to block nonspecific bonds. The slides were incubated with rabbit polyclonal antibody against cytokeratin 17 (1:200, #12509, Cell Signaling Technology Inc.) for overnight at 4 °C. After a reaction was induced through the use of a polymeric technique, the antigen–antibody complex was exposed by incubation to chromogen 3.3′- diaminobenzidine. The cellular nuclei were stained with hematoxylin. Negative controls (without primary antibody) were included.

Quantitative RT-PCR: Total RNA from tissues was extracted using Trizol (Life Technologies, St. Paul, MN) following the manufacturer’s instruction. NanoDrop spectrophotometer method and gel electrophoresis was used to check quantity and quality of RNA. mRNA was quantified with real-time quantitative PCR (QuantStudio 3, Thermo Fisher Scientific) by using one-step RT-PCR Kit (Bio Rad, Hercules, CA) with *18s* as reference genes in each reaction for mouse tissue. The primer and probe sequences are listed in Supplementary Table S1.

Statistical analysis: The sample size was determined by power calculation based on our previous study (α level: 0.05; power: 80%) (Wang et al., 2025). Data are presented as mean ± SEM. Shapiro–Wilk test and Levene’s test were performed to check normality and homogeneity of variance respectively. One way ANOVA test was conducted and Tukey–Kramer HSD test was used for *post hoc* comparison when F is less than 0.05 unless otherwise indicated. All tests were used with the program JMP 17.2.0 (SAS Institute Inc. Cary, NC). Differences were considered to be statistically significant with p values less than 0.05 and exact p value indicated in the figures.

## Results

### Decrease in the implantation site volume in UNx dams is prevented by L-kynurenine/NAM treatment

**FIGURE 1:**
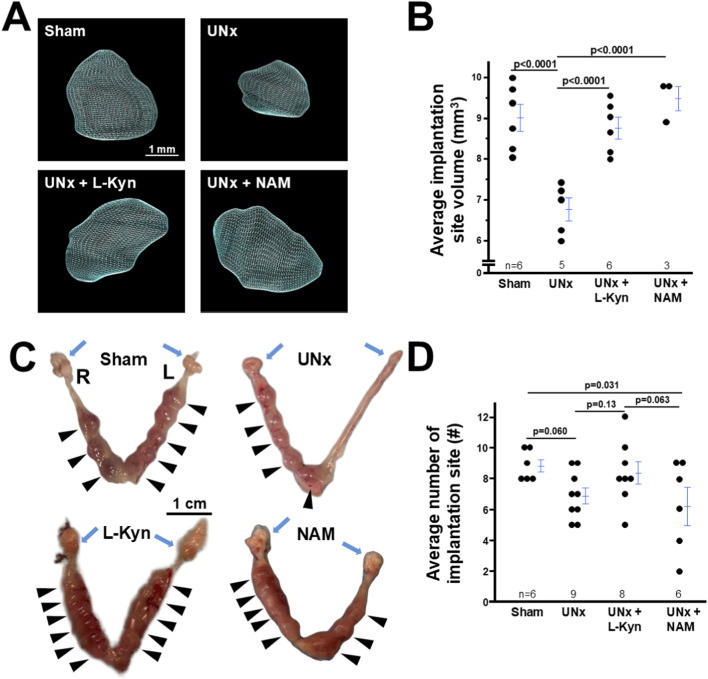
Both L-kyneurine (L-Kyn) and Nicotinamide (NAM) increases the size of implantation sites from dams with uninephrectomy (UNx) at 7.5 dpc. **(A)** Representative 3D visual reconstruction of implantation sites from four groups of mice. **(B)** Average implantation site volume from four groups of dams. 3D volume reconstruction calculations demonstrated the volume of implantation sites of UNx dams. Each point indicated the average volume of the all implantation sites detected in an individual dam. **(C)** L-Kyn and NAM improved the spacing with L-Kyn or NAM of implantation sites of UNx dams. Images of embryos within uteri. R: rihgt uterine horn, L: left uterine horn. Blue arrows: ovaries. Arrow head: viable implantation sites. **(D)** The average number of implantation sites. L-Kyn increased the implantation site number in UNx dams.

Implantation site volume reflects the embryo’s development (Peavey et al., 2017). 3D HFU analysis was used to determine the implantation site volume of different groups of dams and revealed that the implantation site volume at 7.5 dpc from UNx dams was ∼0.75x smaller than that from sham dams. Both L-kynurenine and NMA exposure rescued the implantation site volume in UNx dams (Figures 1A,B). Because prior study showed that neither L-kynurenine nor NMA had effects on normal pregnancy of WT mice (Dupont et al., 2022; Wang et al., 2025; Li et al., 2016), sham mice were underwent only mock nephrectomy but were not treated with either L-kynurenine or NMA in the current study.

Visually examining the implantation sites from the four groups of dams showed that UNx dams had spacing defects and significantly decreased numbers of implantation sites than sham dams. L-kynurenine/NMA exposure increased the implantation site volume and improved spacing in UNx dams (Figures 1C,D). L-kynurenine increased the implantation site number, while NMA did not (Figures 1C,D).

### The impaired structure of implantation sites from UNx dams is improved by L-kynurenine/NAM treatment

**FIGURE 2:**
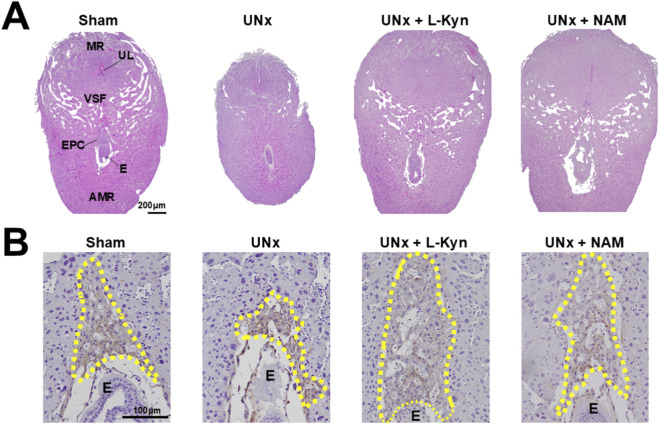
Both L-kyneurine (L-Kyn) and Nicotinamide (NAM) improves the altered morphology of implantation sites from UNx dams at 7.5 dpc. **(A)** Representative images of implantation sites from four groups of dams. H&E staining. Note: Decreased blood vessels in UNx dams. **(B)** Cytokeratin 17 (a marker for trophoblast cells) immunostaining revealed UNx dans had a blunt and irregular invasion of ectoplacental cone cells (enclosed in dashed yellow line) in comparison with sham dams having trophoblast cells sharply invading. Both L-kyneurine (L-Kyn) and Nicotinamide (NAM) corrected this abnormality in UNx dams. E: embryo.

H&E staining of implantation sites from UNx dams at 7.5 dpc showed a reduced vascular sinus folding (VSF) region in the mesometrial region (MR) compared with those from sham dams (Supplementary Figure S1 illustrates the structure of an implantation site). Both L-kynurenine and NAM exposure improved the altered decidual vascular structure of UNx dams (Figures 2A,B).

Embryos from UNx dams had distorted ectoplacental cones (EPCs), and L-kynurenine/NAM exposure corrected its orientation (Figure 2B).

These results suggest that both L-kynurenine and NAM exposure improves the structure of maternal-embryo interface in early pregnancy.

### The decreased expression of decidual vascular endothelial growth factor-A (VEGF) in UNx dams is mitigated by L-kynurenine/NAM treatment

Decreased decidual vascular density suggests that UNx dams have poor angiogenesis. Because VEGF is a crucial factor for decidual angiogenesis in early pregnancy (Kim et al., 2013), we measure decidual VEGF levels in four groups of dams.

**FIGURE 3:**
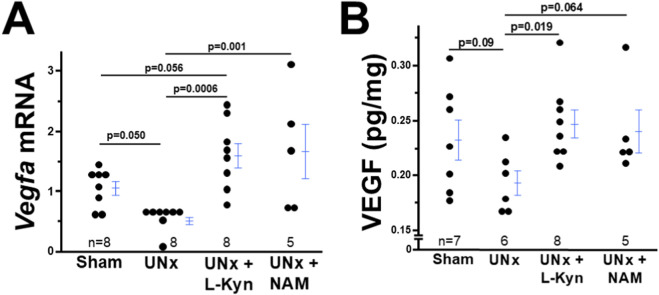
Both L-kyneurine (L-Kyn) and Nicotinamide (NAM) elevates decreased vascular endothelial growth factor A (VEGF) in deciduae from UNx dams at 7.5 dpc. **(A)** The mRNA levles of *Vegfa* in deciduae from four groups of dams. **(B)** The ptptein levels of VEGF in deciduae from four groups of dams.

mRNA levels of *Vegf* in decidual tissues from UNx dams markedly decreased compared with sham dams, in contrast, exposure to L-kynurenine or NAM significantly increased the decidual mRNA levels of Vegf in UNx dams (Figure 3A). In addition, protein levels of decidual VEGF levels determined by ELISA showed the same pattern as mRNA: decidual VEGF was decreased in UNx dams compared with sham dams, and exposure to L-kynurenine or NAM significantly increased the decidual VEGF levels in UNx dams (Figure 3B).

### The decreased expression of markers of decidualization in UNx dams is mitigated by L-kynurenine/NAM treatment

**FIGURE 4:**
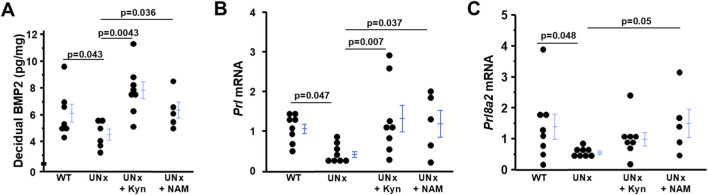
Both L-kyneurine (L-Kyn) and Nicotinamide (NAM) elevates decreased markers of decidualization from UNx dams at 7.5 dpc. **(A)** The amount of BMP2 (bone morphogenetic protein 2). The mRNA levels of **(B)** *Prl* (prolactin) and **(C)** *Prl8a2* (prolactin family 8 subfamily a member 2) in deciduae from four groups of dams. n = 5-8.

Decidualized endometrial stromal cells contribute to angiogenesis, and angiogenesis is essential for decidualization (Matsumoto and Sato, 2006; Zambuto et al., 2024). Accordingly, we investigated the effects of UNx on uterine decidualization. BMP2 (bone morphogenetic protein 2, a marker of endometrial decidualization), determined by ELISA, was reduced in the deciduae of UNx dams than WT dams. L-kynurenine or NAM treatment increased its expression in UNx (Figure 4A).

In addition, the decidual mRNA levels of *Prl* (prolactin) and *Prl8a2* (prolactin family 8 subfamily a member 2) were decreased in UNx dams and L-kynurenine or NAM treatment increased their expression (Figures 4B,C).

### The decrease in progesterone levels in UNx dams is not affected by L-kynurenine/NAM treatment

**FIGURE 5:**
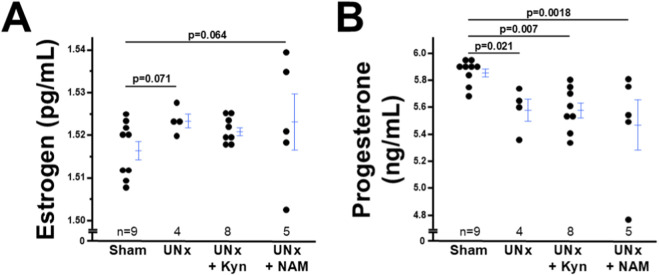
The plasma progesterone levels decreased in UNx dams at 7.5 dpc. **(A)** Plasma estrogen levels. **(B)** Plasma progesterone levels. Note: While UNx significantly decreased progestrone levels, L-Kyneurine (L-Kyn) or Nicotinamide (NAM) did not alter either estrogen or progesterone levels.

Progesterone plays a pivotal role in endometrial decidualization (Okada et al., 2018; Ramathal et al., 2010). We measured plasma levels of progesterone and found that UNx dams had lower circulating progesterone levels than sham dams. Neither L-kynurenine nor NAM increased progesterone levels in UNx dams (Figure 5A). We measured plasma levels of estrogen as well, because it plays a role in stromal cell differentiation (Kaya Okur et al., 2016). The circulating levels of estrogen were not different between UNx and sham dams. Neither L-kynurenine nor NAM altered estrogen levels (Figure 5B).

### The decreased expression of ET-1 and its type B receptor in UNx dams is upregulated by L-kynurenine/NAM treatment

**FIGURE 6:**
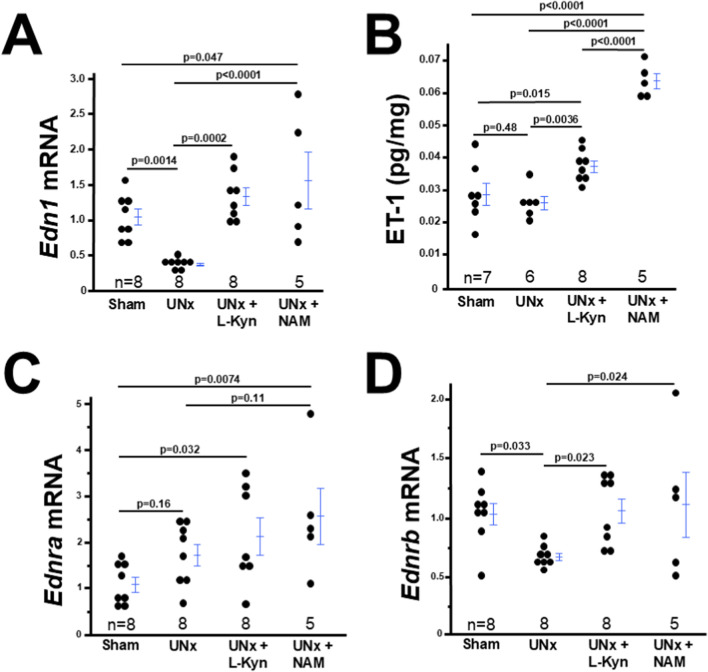
Both L-kyneurine (L-Kyn) and Nicotinamide (NAM) alters the expression of ET-1, and EDNRB in the decidua from UNx dams at 7.5 dpc. **(A)** Decidual *Edn1* mRNA levels in four groups of mice. **(B)** The amount of ET-1 in deciduae from four groups of dams. **(C,D)** Decidual mRNA levels of *Ednra* and *Ednrb* in four groups of mice.

Subtotal nephrectomy led to increased urinary excretions of ET-1 (Ushijima et al., 2017) and our prior study demonstrated that excess ET-1 caused impaired decidualization (Wang et al., 2025). Therefore, we examined the ET-1 levels in the deciduae and circulation. Surprisingly, we found the decidual mRNA levels of *Edn1* significantly decreased in UNx dams compared with sham dams, and L-kynurenine or NAM treatment increased its expression (Figure 6A). The decidual and plasma ET-1 levels had the same pattern as its mRNA levels (Figure 6B; Supplementary Figure S2). Next, we determined the expression of *Ednra* and *Ednrb* and found that the decidual expression of *Ednra* was not altered in UNx dams (Figure 6C). In contrast, the decidual *Ednrb* expression significantly decreased in UNx dams compared with sham dams. L-kynurenine or NAM exposure increased the decidual *Ednrb* expression in UNx dams (Figure 6D).

## Discussion

In the current study, we demonstrated that the impaired renal reserve induced by UNx caused impaired endometrial decidualization and angiogenesis, evidenced by the decreased decidual expression of markers of decidualization and vascular density and VEGF expression during early pregnancy when implantation/invasion occurs. L-kynurenine/NAM treatment mitigated these detrimental effects observed in dams having one kidney. In addition, UNx dams had decreased ET-1 and its type B receptor, both L-kynurenine and NAM treatment increased ET-1 and *Ednrb* in these UNx dams.

The impaired trophoblast cell invasion in early pregnancy, leading to poor placentation is though as the “root” of PE at least for a subset of this disease: early onset PE (Liu et al., 2024). The insufficient endometrial decidualization causes impaired trophoblast cell differentiation and invasion and PE (Garrido-Gomez et al., 2022; Garrido-Gomez et al., 2017). Because female kidney donors have increased risk for PE (Garg et al., 2015; Ibrahim et al., 2009; Reisaeter et al., 2009) and pregnant mice with one kidney develop the PE-like phenotype (Dupont et al., 2022), it suggests the maternal pre-existing condition (e.g., impaired renal reserve) plays an important role in the pathogenesis of PE. It is highly possible that this maternal pre-existing condition affects the endometrial decidualization and maternal-embryo interaction, and later manifestation of PE-like phenotype present in UNx dams. Thus, in the current study, we focused on whether the “soil” (the maternal decidua) was adversely affected by UNx. Indeed, our findings show that inadequate decidualization and angiogenesis were present in UNx dams in early pregnancy when trophoblast cell invasion occurs, suggesting that the early maternal decidualization problem results in the PE-like symptoms in later pregnancy.

Numerous studies including ours have shown that various kidney damage results in elevated ET-1 (De Miguel et al., 2016), including viral infection (Abraham et al., 2022), chronic kidney disease (Zhou et al., 2025) and chronic renal failure (Lariviere and Lebel, 2003), and most relevant to our current study: nephrectomy (Ushijima et al., 2017). Unexpectedly, we found that ET-1 was not higher even lower in UNx dams compared with sham dams, suggesting that low ET-1 plays a role in the pathological changes in implantation sites observed in UNx mice. Indeed, exposure to L-kynurenine/NAM increased ET expression accompanied with improved decidualization process in UNx dams. Our prior study has demonstrated that ET-1 and its two receptors are upregulated during endometrial stromal cell differentiation (decidualization) *in vitro*, and excess ET-1 caused insufficient decidualization in both *in vivo* and *in vitro* models (Wang et al., 2025). Taken together, our data suggests that balanced ET-1 system is critical for normal decidualization, decidual angiogenesis, and maternal-embryo interaction. While the role of excess ET-1 in PE has been studied for many years by investigators around world including us (Li et al., 2018; Li et al., 2012), (Murphy et al., 2010), the reports of the role of lower-than-normal levels of ET-1 in PE are rare. There is one study that showed downregulation of EDN1 gene expression by circulating miR-206 is associated with risk of preeclampsia (Sheng et al., 2020). The role of ET-1 signaling in PE is more complicated than we thought and identifying subsets of preeclamptic women with different levels of ET-1 could help clinicians to provide a tailored intervention strategy.

However, the mechanism of low ET-1 in UNx dams observed in the current study is not clear. The compensatory effect of the contralateral kidney could play a role. For example, nitric oxide (NO) production rises following UNx (Huskic et al., 2006), and NO inhibits ET-1 release (Rapoport, 2014).

NAM is a byproduct of L-kynurenine metabolism (Fukuwatari and Shibata, 2013) and our recent work demonstrates that exposure to NAM improves the decidualization which is impaired by excess ET-1 in both *in vivo* and *in vitro* (Wang et al., 2025). Here, we broaden the beneficial role of NAM beyond excess ET-1 context. Exposure to L-kynurenine or NAM has beneficial effects, and the mechanisms of the effects could overlap between these two small molecules, however, each could also have its only distinct mechanism(s). The shared mechanism could be influencing ET-1 system which is altered by UNx because both L-kynurenine and NAM increased the expression of ET-1 and Ednrb which was suppressed in Unx dams, suggesting that the beneficial role of L-kynurenine/NAM is through ET-1/Ednrb signaling at least partially. Interestingly, this is opposite to our prior fundings that NAM decreased the decidual expression of ET-1 and Ednrb in dams over-expressing ET-1 (Wang et al., 2025). Collectively, these data suggest that NAM could have bidirectional effects, namely, when maternal ET-1 system is over-activated, NAM could suppress the system, however, when the ET-1 system is inhibited, NAM could enhance it. The mechanisms underlying this phenomenon are needed to elucidate.

We observed that the beneficial effects of L-kynurenine are superior to NAM including normalizing the number of implantation sites, increase more in markers of decidualization, suggesting it has its own specific the expression of mechanisms of the beneficial effects which NAM does not have. Because L-kynurenine increased the number of implantation sites what is the effect NAM does not have, we speculate that L-kynurenine improved luminal epithelial cell receptivity of embryo. Notably, in our study, L-kynurenine improved not only the implantation rate but also the spacing between embryos *in utero*. How L-kynurenine exerts these beneficial effects is not known. Future studies are needed to elucidate this important function because it can broaden our understanding of embryo–uterine interactions.

In summary, our current study demonstrated that impaired renal reserve induced by UNx inhibits endometrial decidualization during early stage of pregnancy when embryo implantation/invasion occurs. Both L-kynurenine and NAM treatment improve decidualization and angiogenesis and enhance ET-1/EDNRB signaling. L-kynurenine or NAM supplementation has a potential to improve decidualization and subsequent embryo implantation which could lead to improved pregnancy outcomes, especially pregnancy complications associated with implantation problems including PE.

## Data Availability

The original contributions presented in the study are included in the article/Supplementary Material, further inquiries can be directed to the corresponding author.
