# Supplementary material for: L-kynurenine or nicotinamide supplementations mitigate uterine decidualization impairments during early pregnancy of uninephrectomized mice
Source: Front Physiol. 2026 Mar 10;17:1774244. doi: 10.3389/fphys.2026.1774244 (PMC13008688; doi:10.3389/fphys.2026.1774244)
Supplement: Supplementary file 1 [file DataSheet1.pdf]

Supplemental Information

L-Kynurenine or nicotinamide supplementations mitigate uterine decidualization impairments during early pregnancy of uninephrectomized mice

Yuye Wang , Qing Ma, Meitong Chen, Yukako Kayashima, Nobuyo Maeda-Smithies and Feng Li*

Department of Pathology and Laboratory Medicine, The University of North Carolina, Chapel Hill, NC 27599, USA.

*Correspondence to: Feng Li Ph.D., Department of Pathology and Laboratory Medicine, The University of North Carolina, Chapel Hill, NC 27599, USA.

Phone: 919-597-0864. Fax: 919-966-8800.

E-mail: lif@med.unc.edu

Table S1. Primers and probes for qRT-PCR

Gene	Type	Sequence (5’-3’)
Edn1	Forward	CAG CAG TTA GTG AGA GGA AG
	Reverse	GAC GCT GTT TCT CAT GGT CT
	Probe	FMA-TC CCG AGC GCG TCG TAC CGT ATG- TAMRA
Ednra	Forward	TCT CTG CAA GCT GTT CCC CT
	Reverse	AGC CAC TGC TCT GTA CCT GT
	Probe	FAM-CC TGC AGA AGT CCT CCG TGG GC- TAMRA
Ednrb	Forward	TGG CCA TTT GGA GCT GAG AT
	Reverse	CAG CTC GAT ATC TGT CAA TAC
	Probe	FAM-TG TAA GCT GGT GCC CTT CAT ACA GAA GGC- TAMRA
Prl (Prolactin)	Forward	CTG GCT ACA CCT GAA GAC AA-3′
	Reverse	CAC CAA ACT GAG GAT CAG GT-3′
	Probe	FAM-ACA AGC CCT GAA AGT CCC TCC GGA-Tamra-3′
Prl8a2	Mm00494563_m1	
Vegfa	Forward	CGG TTT AAA TCC TGG AGC GT
	Reverse	ACG TCT GCG GAT CTT GGA CA
	Probe	FAM-CT GTG AGC CTT GTT CAG AGC GGA G- TAMRA
18s	Forward	AGA AAC GGC TAC CAC ATC CA
	Reverse	CTC GAA AGA GTC CTG TAT TGT
	Probe	FAM-AG G CAG CAG GCG CGC AAA TTA C—TAMRA

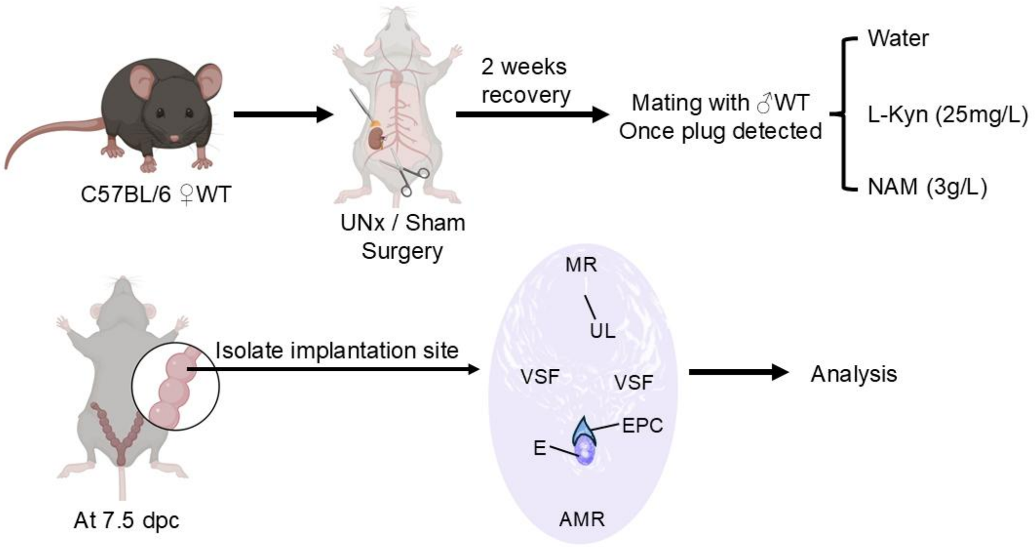

Supplemental Figure 1. Schematic representation of the experimental design. The embryo and ectoplacental cone were carefully removed and the remaining maternal decidua was subjected to either RAN isolation or homogenization in 0.5 ml buffer (0.1% Triton in PBS) for further Western blot/ELISA assay as describe in the **Materials and Methods** section. MR: mesometrial region, UL: uterine lumen, VSF: vascular sinus folding, EPC: ectoplacental cone; E: embryo, AMR: anti-mesometrial region.

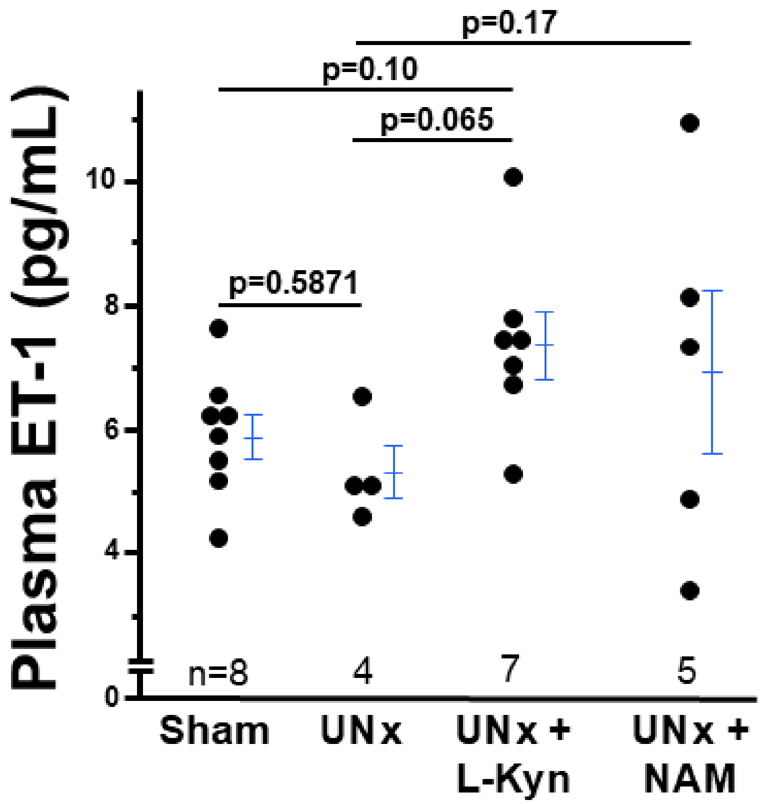

Supplemental Figure 2. Plasma ET-1 concentration at 7.5 dpc from four groups of mice.
